# Partial thioamide scan on the lipopeptaibiotic trichogin GA IV. Effects on folding and bioactivity

**DOI:** 10.3762/bjoc.8.129

**Published:** 2012-07-24

**Authors:** Marta De Zotti, Barbara Biondi, Cristina Peggion, Matteo De Poli, Haleh Fathi, Simona Oancea, Claudio Toniolo, Fernando Formaggio

**Affiliations:** 1 ICB, Padova Unit, CNR, Department of Chemistry, University of Padova, 35131 Padova, Italy; 2 Department of Biochemistry and Toxicology, Lucian Blaga University of Sibiu, 550012 Sibiu, Romania

**Keywords:** bioactivity, conformation, peptaibiotic, peptide synthesis, peptides, thiopeptides

## Abstract

Backbone modification is a common chemical tool to control the conformation of linear peptides and to explore potentially useful effects on their biochemical and biophysical properties. The thioamide, ψ[CS-NH], group is a nearly isosteric structural mimic of the amide (peptide) functionality. In this paper, we describe the solution synthesis, chemical characterization, preferred conformation, and membrane and biological activities of three, carefully selected, peptide analogues of the lipopeptaibiotic [Leu^11^-OMe] trichogin GA IV. In each analogue, a single thioamide replacement was incorporated. Sequence positions near the N-terminus, at the center, and near the C-terminus were investigated. Our results indicate that (i) a thioamide linkage is well tolerated in the overall helical conformation of the [Leu^11^-OMe] lipopeptide analogue and (ii) this backbone modification is compatible with the preservation of its typical membrane leakage and antibiotic properties, although somewhat attenuated.

## Introduction

Since their first incorporation into peptides [1], backbone amide surrogates have attracted remarkable attention from organic and medicinal chemists. Not only may these modifications impart to a peptide an increased resistance to enzymatic hydrolysis as well as higher receptor affinity and specificity, but they may also influence its preferred secondary structure.

A ψ[CS-NH] thioamide group is one of the closest mimics of an amide (peptide) linkage. However, it exhibits significantly different chemical and physical properties, some of which are of great potential interest to peptide chemists [2–44]. Among these properties, we highlight the following: (i) The thioamide NH group is more acidic than that of its oxygenated counterpart and consequently it is a stronger H-bonding donor. (ii) Its *cis*/*trans* isomerization can be phototriggered by irradiation at about 260 nm [absorption maximum of the -C(=S)NH- π→π* electronic transition]. (iii) It may act as a minimalist, effective quencher for any type of protein and nonprotein fluorophores.

**Figure 1:**
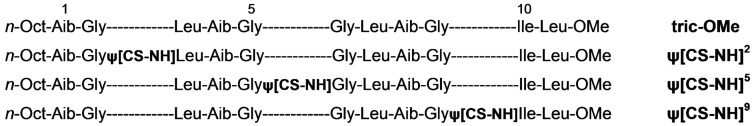
List of primary structures and abbreviations for the peptides studied in this work. The [Leu^11^-OMe] trichogin GA IV is termed **tric-OMe** and its thionated derivatives at position 2, position 5, and position 9 are termed **ψ[CS-NH]^2^**, **ψ[CS-NH]^5^**, and **ψ[CS-NH]^9^**, respectively.

Recently, based on the aforementioned characteristics of the ψ[CS-NH] thioamide bond, we started a program aimed at exploring how thiopeptide groups may affect folding and the related biophysical/biochemical activities of the membrane-active peptaibiotics [45]. This class of compounds represents a subject of long-standing, relevant, interest to our research group. In this paper, we describe our results on the syntheses by solution methods and characterizations of three thioamide-containing [Leu^11^-OMe] analogues of the short helical lipopeptaibiotic trichogin GA IV [46–50] (Figure 1). We already found that the replacement of the native C-terminal 1,2-amino alcohol leucinol (Lol) by the corresponding α-amino methyl ester (Leu-OMe) alters only slightly the biophysical and biological properties of trichogin GA IV. Conversely, we showed that the N^α^-blocking fatty acyl moiety plays a major role in its membrane permeability and antibiotic activity.

In each analogue, the single ψ[CS-NH] group was strategically incorporated either at an internal position of the amino-acid sequence, **ψ[CS-NH]^5^**, or near each of the two ends, **ψ[CS-NH]^2^** and **ψ[CS-NH]^9^**. Moreover, the preferred conformations of these analogues were investigated by circular dichroism (CD), FT-IR absorption, and NMR. Finally, we carried out fluorescence leakage experiments in model membranes and antibacterial assays on a large set of both Gram-positive and Gram-negative strains.

## Results and Discussion

### Peptide synthesis

**Scheme 1:**
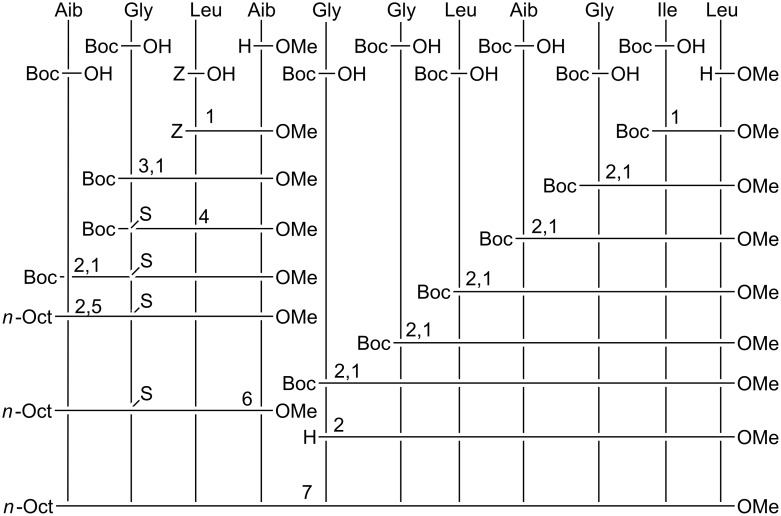
Synthesis of **ψ[CS-NH]^2^**. 1: Coupling in the presence of EDC/HOBt. 2: Deprotection by using TFA/DCM. 3: Deprotection by catalytic hydrogenation with Pd/C. 4: Thionation with Lawesson's reagent in THF. 5: Coupling with *n*-Oct-OH in the presence of EDC/HOBt. 6: Saponification with NaOH/MeOH. 7: Coupling in the presence of EDC/HOAt.

**Scheme 2:**
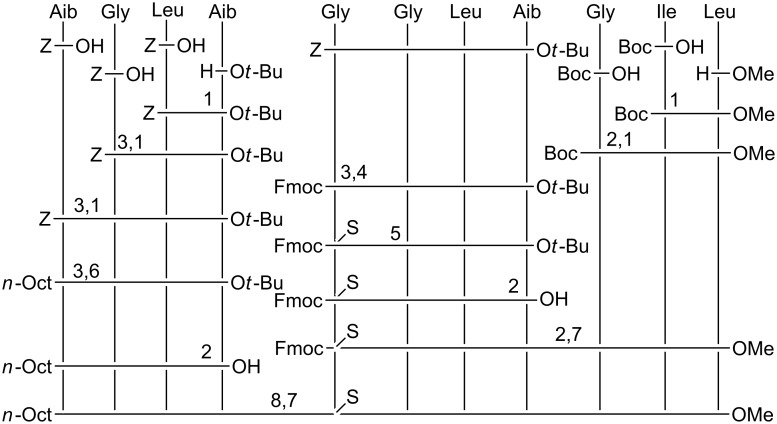
Synthesis of **ψ[CS-NH]^5^**. 1: Coupling in the presence of EDC/HOBt. 2: Deprotection by using TFA/DCM. 3: Deprotection by catalytic hydrogenation with Pd/C. 4: Coupling with Fmoc-OSu in 1,4-dioxane. 5: Thionation with Lawesson's reagent in THF. 6: Coupling with *n*-Oct-OH in the presence of EDC/HOBt. 7: Coupling in the presence of EDC/HOAt. 8: Deprotection with DEA in DCM.

**Scheme 3:**
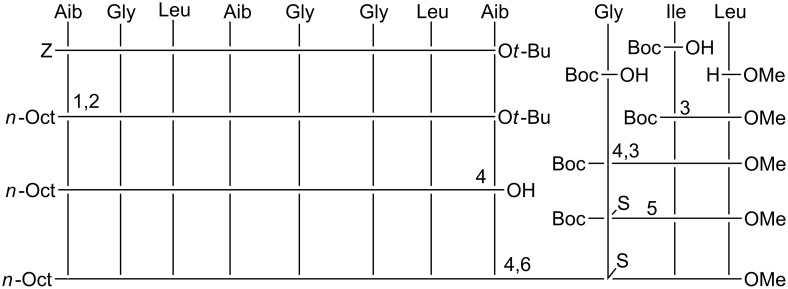
Synthesis of **ψ[CS-NH]^9^**. 1: Deprotection by catalytic hydrogenation with Pd/C. 2: Coupling with *n*-Oct-OH in the presence of EDC/HOBt. 3: Coupling in the presence of EDC/HOBt. 4: Deprotection by using TFA/DCM. 5: Thionation with Lawesson's reagent in THF. 6: Coupling in the presence of EDC/HOAt.

The total syntheses of the three monothionated ψ[CS-NH] trichogin GA IV analogues at positions 2, 5, and 9, respectively, were accomplished by using the solution-phase method (Schemes 1–3). Both step-by-step and segment condensation approaches were utilized. The former strategy was used in the preparation of Boc-Gly^5^-Gly-Leu-Aib-Gly-Ile-Leu^11^-OMe (segment 5–11, for the synthesis of **ψ[CS-NH]^2^**), Boc-Gly^9^-Ile-Leu^11^-OMe (segment 9–11), and *n*-Oct-Aib^1^-Gly-Leu-Aib^4^-O*t*-Bu (segment 1–4, for the synthesis of **ψ[CS-NH]^5^**), and *n*-Oct-Aib^1^-Gly-Leu-Aib-Gly-Gly-Leu-Aib^8^-O*t*-Bu (segment 1–8, for the synthesis of **ψ[CS-NH]^9^**). The latter strategy, which permits a faster preparation of multiple analogues, afforded (i) **ψ[CS-NH]^2^** by condensation of the thionated 1–4 and the 5–11 segments; (ii) **ψ[CS-NH]^5^** by condensation of the thionated 5–8 and the 9–11 segments followed by condensation of the 1–4 and the resulting, thionated, 5–11 segments; and (iii) **ψ[CS-NH]^9^** by condensation of the 1–8 and the thionated 9–11 segments.

For the difficult coupling steps, in particular those involving the segment condensations, the *N*-[3-(dimethylamino)-propyl]-*N*'-ethylcarbodiimide (EDC)/7-aza-1-hydroxy-1,2,3-benzotriazole (HOAt) [51] activating method was used, while the EDC/1-hydroxy-1,2,3-benzotriazole (HOBt) [52] method turned out to be appropriate for the formation of the other peptide bonds. The yields of the segment condensation reactions were good (75–83%). An important feature, which makes trichogin GA IV a versatile synthetic platform, is the presence of as many as seven achiral (Aib or Gly) residues spread across its sequence, which allows one to design suitable segments each with an achiral residue at its C-terminus, thus reducing dramatically the usually considerable risk of epimerization during the coupling reactions.

For the best choice of the step involving treatment with the Lawesson's reagent [53], we decided to select the longest segment of our sequences, which, in principle, could permit complete regioselectivity between the peptide bond to be thionated and the other peptide bonds (in addition to the known regioselectivity versus the urethane and ester bonds) [53–56]. In this reaction, regioselectivity is known to be heavily governed by the steric hindrance of the two residues around the peptide bond. Therefore, we synthesized Boc-Gly^2^ψ[CS-NH]Leu-Aib^4^-OMe (for the preparation of **ψ[CS-NH]^2^**), Fmoc-Gly^5^ψ[CS-NH]Gly-Leu-Aib^8^-O*t*-Bu (for the preparation of **ψ[CS-NH]^5^**), and Boc-Gly^9^ψ[CS-NH]Ile-Leu^11^-OMe (for the preparation of **ψ[CS-NH]^9^**). Thionation of a peptide as short as a dipeptide is not recommended because a further synthetic step at the C-terminus of this compound would not take place satisfactorily, owing to the easy formation of a poorly reactive 1,3-thiazolidin-5-one. All thionation steps were conducted by use of the Lawesson's reagent under mild conditions (in tetrahydrofuran at room temperature) [53–56]. The only synthetic problem was identified in the preparation of the Fmoc-Gly^5^ψ[CS-NH]Gly-Leu-Aib^8^-O*t*-Bu, in which case the thionation selectivity on the Gly-Gly peptide bond was incomplete. Indeed, a limited production (about 10%) of the isomeric Fmoc-Gly-Gly^6^ψ[CS-NH]Leu-Aib-O*t*-Bu, with its slightly more hindered, thionated Gly-Leu peptide bond, was observed. In this case, the desired monothionated peptide was separated from its isomer by means of flash chromatography.

**Figure 2:**
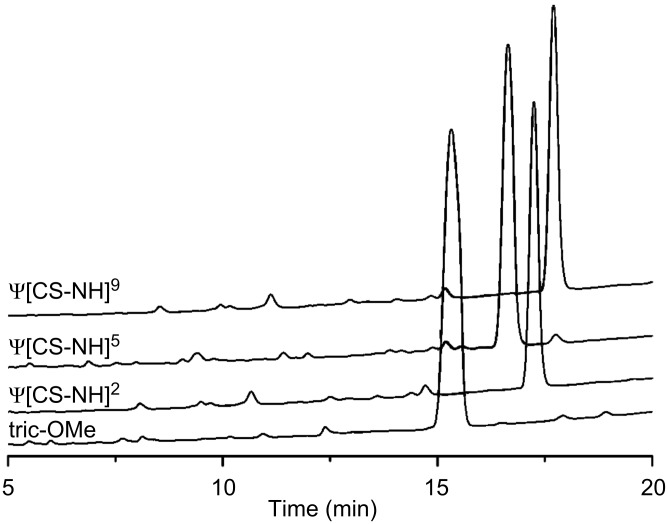
RP-HPLC profiles obtained for [Leu^11^-OMe] trichogin GA IV (**tric-OMe**) and its **ψ[CS-NH]^2^**, **ψ[CS-NH]^5^**, and **ψ[CS-NH]^9^** analogues.

For details of the synthetic procedures and characterizations of these analogues (and the synthetic precursor segments as well), see the Experimental section and Supporting Information File 1. Figure 2 gives the RP-HPLC profiles of the three monothionated trichogin GA IV analogues.

### Conformational analysis

A detailed analysis of the spectroscopic properties and conformational preferences of the three monothionated trichogin GA IV analogues synthesized in this work was performed by using CD, FT-IR absorption, and 2D NMR investigations in different solvents.

**Figure 3:**
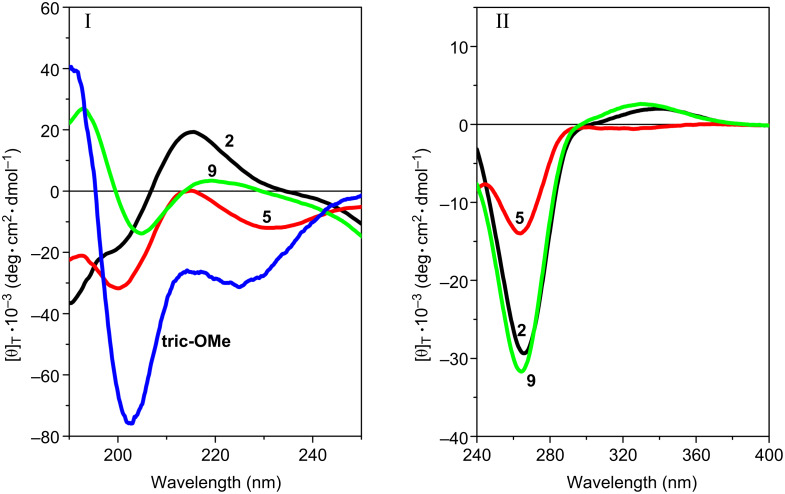
Far-UV (panel I) and near-UV (panel II) CD spectra of [Leu^11^-OMe] trichogin GA IV (**tric-OMe**) and its **ψ[CS-NH]^2^** (**2**), **ψ[CS-NH]^5^** (**5**), and **ψ[CS-NH]^9^** (**9**) analogues in TFE solution. Peptide concentration: 1 mM.

The far- (250–195 nm) and near-UV (400–250 nm) CD spectra of the three peptides, recorded in 2,2,2-trifluoroethanol (TFE) solution, are illustrated in Figure 3. In the near-UV region, in which the peptide chromophore is known not to absorb, both **ψ[CS-NH]^2^** and **ψ[CS-NH]^9^** display two well-separated Cotton effects at about 335 and 265 nm. The longer wavelength band is positive and weak, while the shorter wavelength band is negative and much more intense. Interestingly, the positive band is absent and the negative band is much weaker in **ψ[CS-NH]^5^**. The spectral positions of these Cotton effects correspond to a very intense maximum (265 nm) and an extremely weak maximum (320 nm) found in the corresponding region of the UV absorption spectra (not shown) of our peptides in the same solvent. We assign the two bands to the π→π* and n→π* transitions, respectively, of thioamide chromophore [29–30,32–36,40–41,43]. We attribute the overall much less intense CD spectrum of **ψ[CS-NH]^5^** in this region primarily to a thioamide chromophore positioned in this peptide between the two achiral Gly^5^ and Gly^6^ residues, i.e., it is significantly far apart from the nearest chiral center (the Leu^7^ α-carbon).

In the far-UV region the CD spectra of all three analogues show multiple (negative/positive) Cotton effects of weak and medium intensities. Here, the bands (shoulders) of **ψ[CS-NH]^9^** are the weakest. In our view, it is risky to attempt to extract any conclusive conformational information from the CD properties of these three isomeric peptides between 195 and 250 nm because, in addition to the expected Cotton effects of the peptide chromophores [57–58], the thioamide chromophore [34–35] is also known to contribute heavily in this spectral region. However, we can safely state that the shape of the CD spectrum of the [Leu^11^-OMe] trichogin GA IV prototypical peptide [50,59] is most closely paralleled by that of its **ψ[CS-NH]^5^** analogue, although the former is significantly more intense. This finding is not surprising in view of the above-mentioned low influence of the thioamide chromophore on the spectrum of this monothionated compound. It may also suggest that the overall conformation of the lipopeptaibiotic is disturbed by this specific oxygen-to-sulfur exchange only to a limited extent.

**Figure 4:**
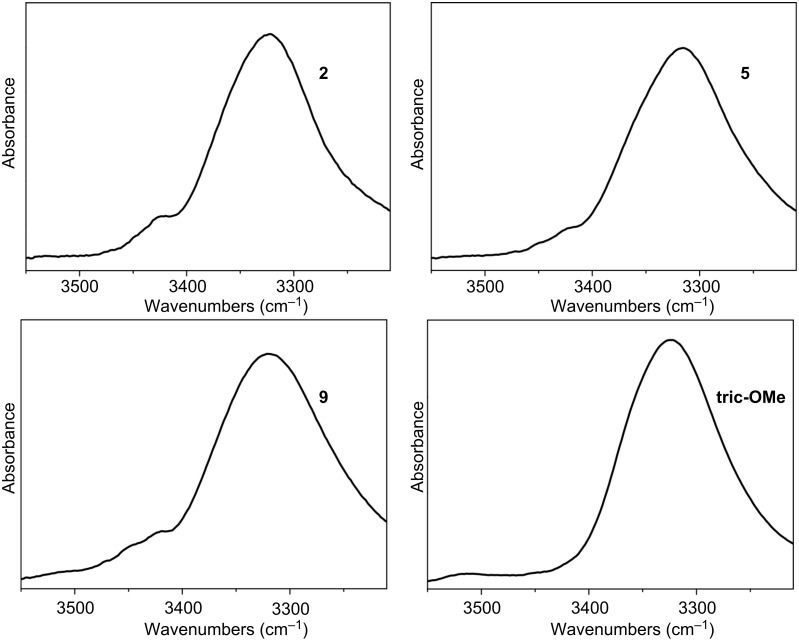
FT-IR absorption spectra (3550–3200 cm^−1^ region) in CDCl_3_ solution of [Leu^11^-OMe] trichogin GA IV (**tric-OMe**) and its **ψ[CS-NH]^2^** (**2**), **ψ[CS-NH]^5^** (**5**), and **ψ[CS-NH]^9^** (**9**) analogues. Peptide concentration: 1 mM.

The preferred conformations of the three monothionated [Leu^11^-OMe] trichogin GA IV analogues were more safely analyzed by means of FT-IR absorption and 2D NMR. In this study, we used CDCl_3_, a solvent of low polarity. In the 3D-structurally informative 3550–3200 cm^−1^ region at 1.0 mM concentration, the FT-IR spectra of [Leu^11^-OMe] trichogin GA IV and its analogues are similar and dominated by a broad and intense absorption at 3316–3328 cm^−1^ (Figure 4), assigned to the N–H stretching mode of the largely prevailing H-bonded peptide groups [60–61]. Additional, very weak bands are visible in the 3450–3400 cm^−1^ region, mostly attributed to free (solvated) peptide NH groups.

A nonnegligible dilution effect is observed in the spectra of the four peptides between 1.0 and 0.1 mM concentration (not shown). We ascribe this difference to the well-known tendency of trichogin GA IV and its analogues to self-associate above 1.0 mM concentration in CDCl_3_ solution. We interpret the spectra at 0.1 mM concentration as arising almost exclusively from intramolecular C=O(S)···H–N interactions. It is clear that under these experimental conditions the preferred conformation of [Leu^11^-OMe] trichogin GA IV [46–50] and its three analogues, all rich in the helix-supporting Aib residues, is highly folded and largely stabilized by intramolecular H-bonds. In the amide I (C=O stretching) region, the maximum of the absorption band of the four peptides is located between 1662 and 1656 cm^−1^ (not shown), close to the canonical positions of this band [61–62] in α- and 3_10_-helical peptides [63].

**Figure 5:**
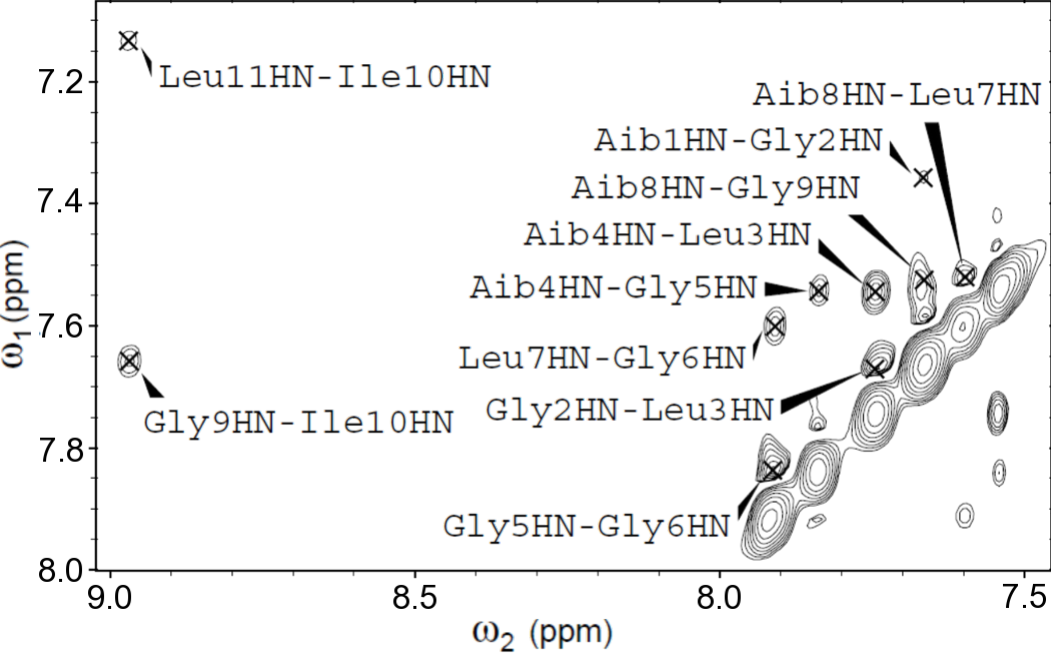
Region of the amide NH protons in the H/H-ROESY spectrum of **ψ[CS-NH]^9^** (400 MHz, 1 mM in CD_3_CN solution, 298 K).

**Figure 6:**
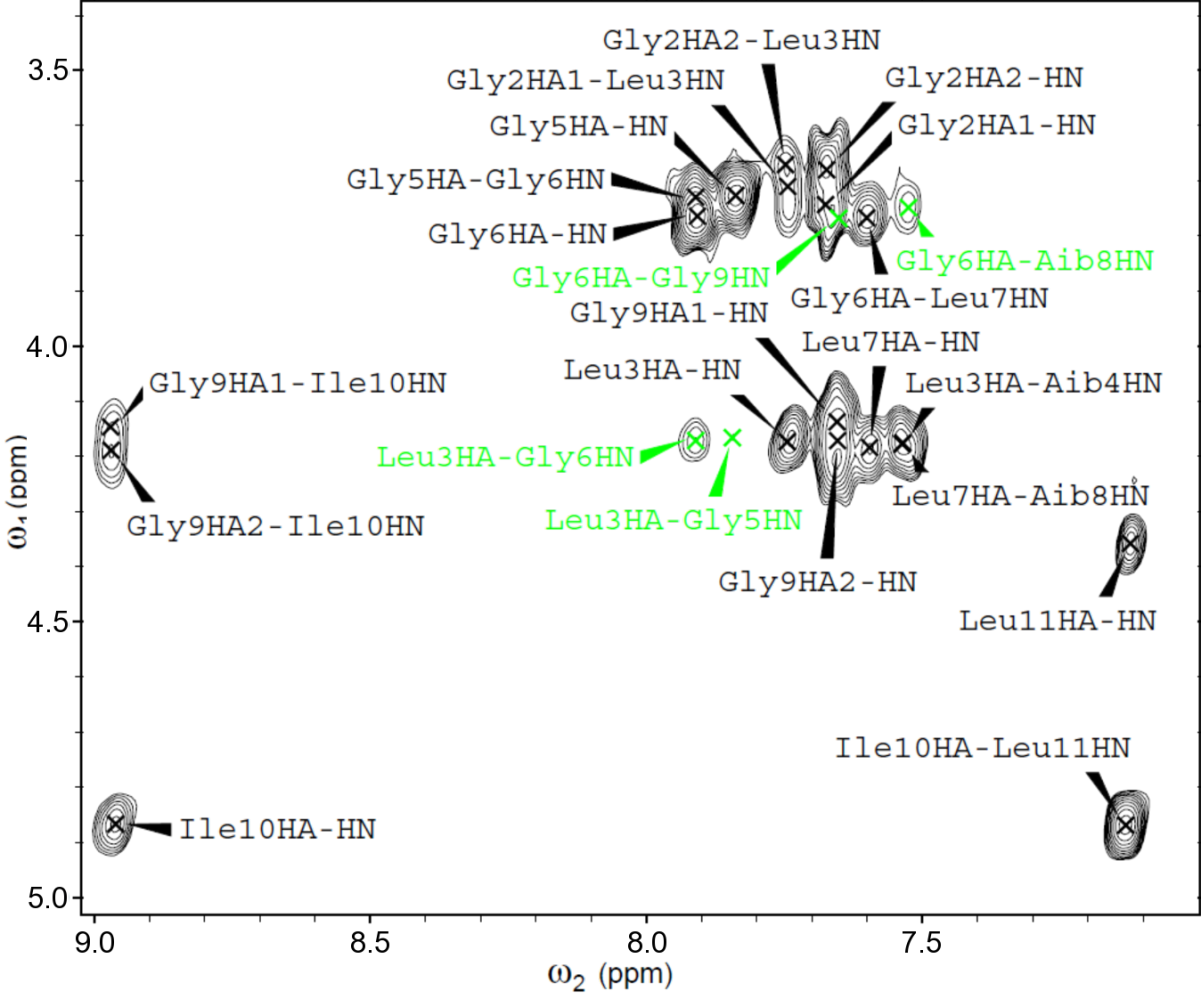
Fingerprint region of the H/H-ROESY spectrum of **ψ[CS-NH]^9^** (400 MHz, 1 mM in CD_3_CN solution, 298 K). The C^α^H*_i_* → NH*_i_*_+2_, and C^α^H*_i_* → NH*_i_*_+3_ cross peaks are highlighted in color.

**Figure 7:**
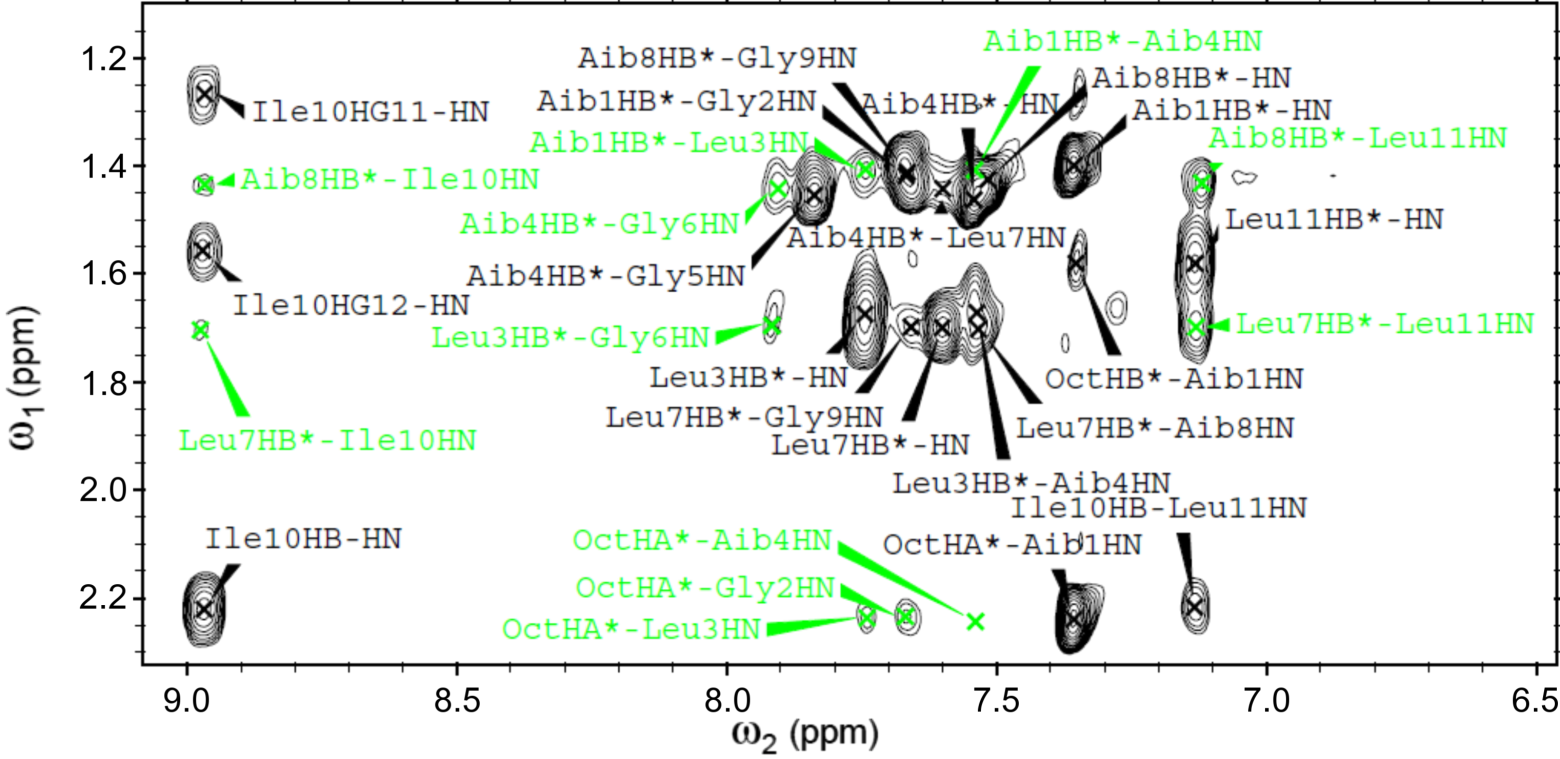
Fingerprint region of the H/H-ROESY spectrum of **ψ[CS-NH]^9^** (400 MHz, 1 mM in CD_3_CN solution, 298 K). The C^β^H*_i_* → NH*_i_*_+2_, C^β^H*_i_* → NH*_i_*_+3_, and C^β^H*_i_* → NH*_i_*_+4_ cross peaks are highlighted in color.

A 2D NMR investigation was performed in CD_3_CN solution for all of the ψ[CS-NH]-containing [Leu^11^-OMe] trichogin GA IV analogues. The proton resonances were fully assigned following the Wüthrich procedure [64]. Regions of the ROESY spectrum acquired for **ψ[CS-NH]^9^** are shown in Figures 5–7. The patterns of NH(*i*)–NH(*i*+1) connectivities (e.g., Figure 5) strongly suggest the onset of an overall helical structure for all analogues. More relevant 3D-structural information was extracted from the *fingerprint* regions of the ROESY spectra (e.g., Figure 6 and Figure 7), which show evidence for *d*_α(β),N_ (*i*, *i*+2), (*i*, *i*+3), and (*i*, *i*+4) medium-range connectivities, diagnostic of the presence of mixed 3_10_-/α-helical conformations.

The conformational properties of **ψ[CS-NH]^9^** were further investigated by simulated annealing (SA) and restrained molecular dynamics (MD) calculations. A total of 69 interproton distance restraints, derived from the related ROESY spectrum (Table S-I, Supporting Information File 1), were used in the SA protocol. The structures possessing violations to the NOE restraints lower than 0.5 Å were selected out of the 150 generated structures. The superposition of the 18 structures with a total energy <144 kcal/mol is shown in Figure S-I, Supporting Information File 1. All these structures converge to a well-defined, right-handed, mixed 3_10_-/α-helical conformation throughout the sequence with a backbone average pairwise root-mean-square deviation of 0.49 ± 0.18 Å (deviations from idealized geometry and mean energies for the 18 lowest energy structures are listed in Table S-I, Supporting Information File 1). Due to heavily overlapped ROE signals, the helical structures originated from the MD calculations appear not to superimpose perfectly in correspondence with the first three residues of the sequence. Even with this experimental limit, the residues belonging to the central and C-terminal parts of the sequence showed values of torsion angles corresponding to those characteristic of right-handed helices (Table S-II, Supporting Information File 1). The helical structures are stabilized by intramolecular H-bonds throughout the sequence. H-bonds both of the α- (*i*←*i*+4) and 3_10_- (*i*←*i*+3) helical types are present, although with a large predominance of the latter type (mean distance: 1.81 ± 0.17 Å; mean angle: 27.4 ± 3.7°), at the N-terminus. Conversely, only the former type (mean distance: 1.53 ± 0.02 Å; mean angle: 27.3 ± 2.9°) was detected at the C-terminus.

**Figure 8:**
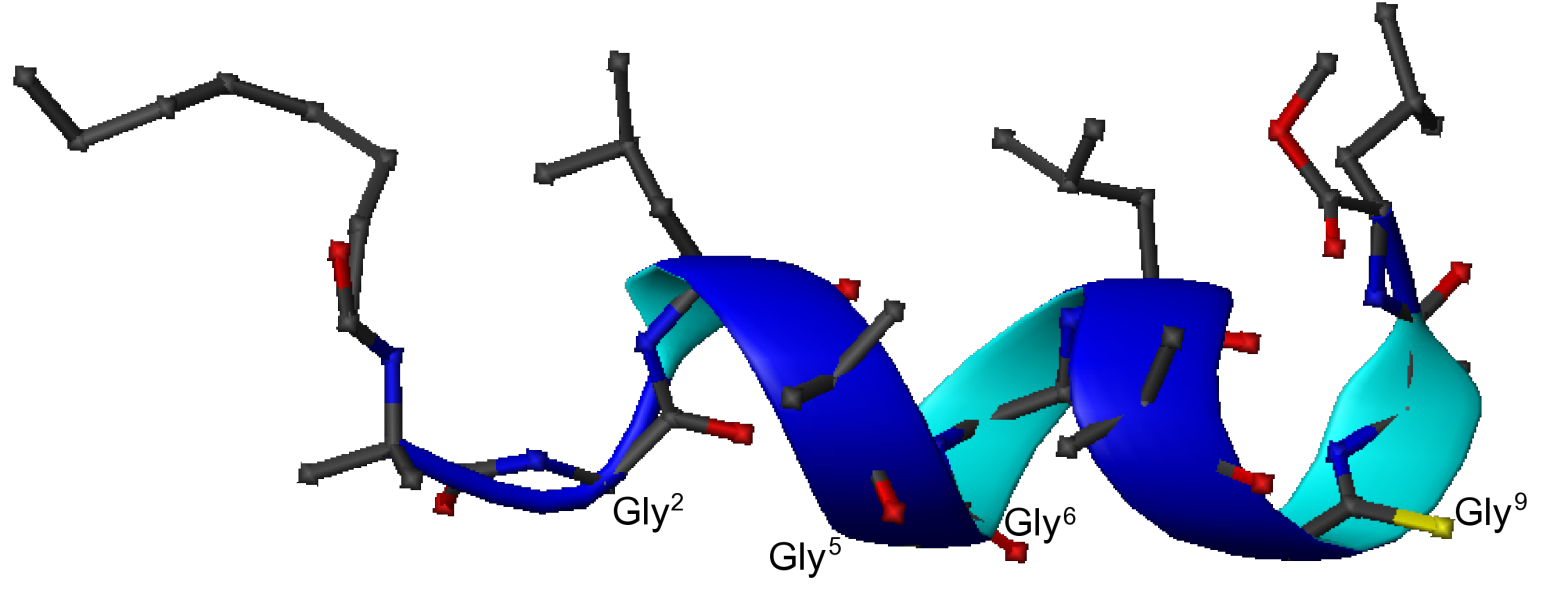
Ribbon representation of the lowest energy (138.7 kcal/mol) 3D structure obtained for **ψ[CS-NH]^9^**. All amino acid side chains are shown. The yellow atom on Gly^9^ refers to sulfur.

In conclusion, the MD calculations based on the restraints derived from the ROESY spectrum of **ψ[CS-NH]^9^** reveal the marked preference of this analogue for a mixed α-/3_10_- helical conformation, with a clear α-helical character at the C-terminus on which the thioamide bond is present. The lowest-energy 3D structure, shown in Figure 8, exhibits the slightly amphipathic character typical of the native peptide, with the poorly hydrophilic Gly residues aligned on the same face of the helix.

### Membrane and biological activities

The membrane permeability properties of [Leu^11^-OMe] trichogin GA IV and its monothionated analogues were tested by measuring the induced leakage of 5(6)-carboxyfluorescein (CF) entrapped in small unilamellar vesicles (SUVs) [65]. For this investigation, the overall negatively charged 1,2-dioleoyl-*sn*-glycero-3-phosphoethanolamine (DOPE)/1,2-dioleoyl-*sn*-glycero-3-phospho-(1'-*rac*-glycerol) (DOPG) model membrane was exploited. The extent of permeation induced by the three analogues is similar, although 30–40% lower than that of the parent peptaibiotic.

The antimicrobial activities were assessed on a large set of Gram-positive and Gram-negative bacterial strains. We found that [Leu^11^-OMe] trichogin GA IV is active on the Gram-positive bacteria *Staphylococcus aureus* and *Streptococcus pyrogenes*, whereas the spectrum of action of its three analogues is more restricted, as they are not active on the latter strain. The activities of the analogues on *S. aureus* are comparable, but reduced by about 40% with respect to that of the parent peptaibiotic.

## Conclusion

In this work, we performed a partial thioamide scan of the short lipopeptaibiotic [Leu^11^-OMe] trichogin GA IV by synthesizing and studying three thioamide-containing analogues. The syntheses were accomplished in solution by using a combination of the step-by-step and segment condensation methods. Appropriate tri- and tetrapeptide segments were monothionated by treating the all-amide precursors with the Lawesson's reagent. Our CD, FT-IR absorption, and NMR conformational investigations show that all analogues maintain the mixed 3_10_-/α-helical structure and the self-association propensity of the native lipopeptide. They also preserve, at least to a great extent, its well-established capability to interact with model phospholipid membranes and to exhibit activity against Gram-positive bacterial strains.

## Experimental

All reagents and solvents, of analytical grade and purchased from commercial sources, were used without further purification. Melting points were measured by means of a capillary tube immersed in an oil bath (Tottoli apparatus, Büchi) and are uncorrected. Optical rotations [α]_D_^20^ (given in units of 10^−1^ deg·cm^2^·g^−1^) were measured at 20 °C on a Perkin-Elmer PE241 polarimeter, with a 1 dm path length cell, at the D-wavelength of sodium (589 nm). The concentration of each compound (*c*) is given in mg/cL. Mass spectroscopy (electrospray ionization, ESIMS) was performed by using a PerSeptive Biosystem Mariner instrument (Framingham, MA). Analytical TLC and preparative column chromatography were performed on Kieselgel F 254 and Kieselgel 60 (0.040–0.063 mm) (Merck), respectively. The retention factor (*R*_f_) values were determined by using three solvent mixtures as eluants: *R*_f1_: chloroform/ethanol 9:1; *R*_f2_: 1-butanol/acetic acid/water 3:1:1; *R*_f3_: toluene/ethanol 7:1.

### General procedure for thionation of peptides

To a solution of the peptide (1 mmol) in anhydrous tetrahydrofuran under nitrogen atmosphere, the Lawesson's reagent (0.6 mmol) was added. The reaction mixture was stirred at rt overnight. After removal of the solvent under reduced pressure, the crude product was purified by flash chromatography with a mixture of ethyl acetate and petroleum ether as eluant.

Peptides Boc-Ile-Leu-OMe [66], Z-Leu-Aib-OMe [67], Z-Leu-Aib-O*t*-Bu [68], Boc-Gly-Leu-Aib-OMe [69], Boc-Gly-Ile-Leu-OMe [66], Boc-Leu-Aib-Gly-Ile-Leu-OMe [66], Boc-Gly-Gly-Leu-Aib-Gly-Ile-Leu-OMe [66], Z-Gly-Gly-Leu-Aib-O*t*-Bu [59], and Z-Aib-Gly-Leu-Aib-Gly-Gly-Leu-Aib-O*t*-Bu [59] are known compounds. Chemical characterizations and selected NMR spectra for the other peptides reported in Schemes 1–3 are given in Supporting Information File 1.

### Circular dichroism

The CD spectra were measured on a Jasco (Tokyo, Japan) model J-715 spectropolarimeter equipped with a Haake thermostat (Thermo Fisher Scientific, Waltham, MA). Baselines were corrected by subtracting the solvent contribution. Fused quartz cells of 1.0 mm and 10.0 mm path lengths (Hellma, Mühlheim, Germany) were used. The values are expressed in terms of [θ]_T_, the total molar ellipticity (deg·cm^2^·dmol^−1^). Spectrograde TFE and 99.9% MeOH (Acros Organic, Geel, Belgium) were employed as solvents.

#### FT-IR absorption

The FT-IR absorption spectra were recorded at 293 K by using a Perkin-Elmer model 1720X FT-IR spectrophotometer, nitrogen-flushed, equipped with a sample-shuttle device, at 2 cm^−1^ nominal resolution, averaging 100 scans. Solvent (baseline) spectra were recorded under the same conditions. For spectral elaboration, the software SPECTRACALC provided by Galactic (Salem, MA) was employed. Cells with path lengths of 1.0 and 10 mm (with CaF_2_ windows) were used. Spectrograde deuterated chloroform (99.8%, *d*) was purchased from Merck (Darmstadt, Germany).

#### Nuclear magnetic resonance

All ^1^H NMR experiments were performed on a Bruker AVANCE DMX-600, DRX-400 or AC200 spectrometer operating at 600, 400 or 200 MHz, respectively, using the TOPSPIN software package. Splitting patterns are abbreviated as (s) singlet, (d) doublet, (t) triplet, (q) quartet, (m) multiplet. The homonuclear 2D spectra of the three monothionated [Leu^11^-OMe] trichogin GA IV analogues were recorded at 298 K, with CD_3_CN as solvent. All spectra were acquired by recording 512 experiments, each one consisting of 64–80 scans and 2000 data points. The spin systems of protein amino acid residues were identified by using standard DQF-COSY [70] and CLEAN-TOCSY [71–72] spectra. In the latter case, the spin-lock pulse sequence was 70 ms long. A ROESY experiment was exploited for sequence-specific assignment. The mixing time of the ROESY experiment acquired for **ψ[CS-NH]^9^** (peptide concentration: 1.00 mM in CD_3_CN) and used for interproton distance determination was 200 ms. Interproton distances were obtained by integration of the ROESY spectra using SPARKY 3.111. The calibration was based on the average of the integration values of the cross peaks due to the interactions between the sequential amide protons, set to a distance of 2.80 Å. When peaks could not be integrated because of partial overlap, a distance corresponding to the maximum limit of detection of the experiment (4.0 Å) was assigned to the corresponding proton pair.

MD calculations were carried out using the SA protocol of the XPLOR-NIH 2.9.6 program [73]. For distances involving equivalent or non-stereo-assigned protons, an r^−6^ averaging was used. The MD calculations involved a minimization stage of 100 cycles, followed by SA and refinement stages. The SA consisted of 30 ps of dynamics at 1,500 K (10,000 cycles, in 3 fs steps) and of 30 ps of cooling from 1,500 to 100 K in 50 K decrements (15,000 cycles, in 2 fs steps). The SA procedure, in which the weights of ROE and nonbonded terms were gradually increased, was followed by 200 cycles of energy minimization. In the SA refinement stage, the system was cooled from 1,000 to 100 K in 50 K decrements (20,000 cycles, in 1 fs steps). Finally, the calculations were completed with 200 cycles of energy minimization by using a NOE force constant of 50 kcal/mol. The generated structures were visualized with the MOLMOL [74] (version 2K.2) program.

#### Small unilamellar vesicles (SUVs) preparation

DOPE and DOPG were purchased from Avanti Polar Lipids, Inc. (Alabaster, AL). The preparation of SUVs was performed in a similar way as described in reference [59].

#### Leakage from lipid vesicles

The peptide-induced leakage from SUVs was measured at 293 K by using the CF-entrapped vesicle technique [65] and a Perkin Elmer model MPF-66 spectrofluorimeter. SUVs were prepared as described above. The phospholipid concentration was kept constant (0.06 mM), and increasing [peptide]/[lipid] molar ratios (*R*^−1^) were obtained by adding aliquots of each non-hydrosoluble, monothionated peptide (or of trichogin GA IV, used as reference compound) as a MeOH solution, keeping the final MeOH concentration below 5% by volume. After rapid and vigorous stirring, the time course of fluorescence change corresponding to CF escape was recorded at 520 nm (6 nm band pass) with λ_exc_ 488 nm (3 nm band pass). The percentage of released CF at time *t* was determined as 100 × (*F*_t_−*F*_0_)/(*F*_T_−*F*_0_), with *F*_0_ = fluorescence intensity of vesicles in the absence of peptide, *F*_t_ = fluorescence intensity of vesicles at time *t* in the presence of peptide, and *F*_T_ = total fluorescence intensity determined by disrupting the vesicles by the addition of 50 μL of a Triton X-100 solution. The experiments were stopped at 20 min.

#### Antibacterial activity

Peptide antibacterial activity was tested against Gram-positive and Gram-negative bacteria by the standardized disk diffusion Bauer–Kirby method [75] using the Müller–Hinton culture medium at pH 7.2–7.4 as recommended by the National Committee for Clinical Laboratory Standards [76]. The antibacterial activity assay was performed in a similar way to that described in reference [77]. The activity of peptides was tested against clinical isolates of bacteria and reference bacterial strains: *Staphylococcus aureus* American Type Culture Collection strains (ATCC) 25923, *Streptococcus pyogenes* ATCC 19615, *Escherichia coli* ATCC 25922, *Pseudomonas aeruginosa* ATCC 27853, *Klebsiella pneumoniae* ATCC 13883, *Salmonella entereditis* 13076, and *Proteus mirabilis* ATCC 10975. The well-known antibiotics bacitracin and tetracyclin (10 μg/disk) were used as controls.

## Supporting Information

File 1 Chemical characterization data for the peptides reported in Schemes 1–3.
