# Supplementary material for: Partial thioamide scan on the lipopeptaibiotic trichogin GA IV. Effects on folding and bioactivity
Source: Beilstein J Org Chem. 2012 Jul 24;8:1161–71. doi: 10.3762/bjoc.8.129 (PMC3458734; doi:10.3762/bjoc.8.129)
Supplement: File 1 — Chemical characterization data for the peptides reported in Schemes 1–3. [file Beilstein_J_Org_Chem-08-1161-s001.pdf]

Supporting Information

for

Partial thioamide scan on the lipopeptaibiotic trichogin GA IV. Effects on folding and bioactivity

Marta De Zotti[1], Barbara Biondi[1], Cristina Peggion[1], Matteo De Poli[1], Haleh Fathi[1], Simona Oancea[2], Claudio Toniolo[1] and Fernando Formaggio*[1]

Address: [1]ICB, Padova Unit, CNR, Department of Chemistry, University of Padova, 35131 Padova, Italy and [2]Department of Biochemistry and Toxicology, Lucian Blaga University of Sibiu, 550012 Sibiu, Romania

Email: Fernando Formaggio* - fernando.formaggio@unipd.it

*Corresponding author

Chemical characterization data for the peptides reported in Schemes 1–3.

Table S-I: ROE constraints, deviations from idealized geometry, and mean energies for the NMR-based structures (energy < 144 kcal/mol) of **ψ[CS-NH][9]**.

Number of NOEs	
total	69
intraresidue	24
sequential	23
i,*i*+*n*, *n* = 2, 3, 4	22
Mean rmsd[(a)] from ideality of accepted structures	
bonds (Å)	0.0086
angles (°)	1.05
impropers (°)	67.0
ROEs (Å)	0.118
Mean energies (kcal/mol) of accepted structures	
$E_{overall}$	140.8
E_{bond}	12.6
E_{angle}	46.7
E_{ROE}	44.3

(a) Root-mean-square deviation.

Table S-II: Average values (°) for the torsion angles ϕ_m and ψ_m and their relative standard deviations resulting from the 18 calculated structures (energy < 144 kcal/mol) of **ψ[CS-NH][9]** (400 MHz, 1 mM in CD_3CN solution, 298 K).

Residue	ϕ_m	$\Delta\phi$	ψ_m	$\Delta\psi$
Aib[1]	—	—	67.1	±1.0
Gly[2]	146.8	±5.3	−33.5	±2.6
L-Leu[3]	−46.2	±2.6	−45.1	±3.2
Aib[4]	−79.5	±1.0	−24.6	±3.2
Gly[5]	–130.2	±4.9	−22.2	±5.0
Gly[6]	−71.8	±2.6	−25.3	±1.7
L-Leu[7]	−74.5	±1.5	−28.1	±3.5
Aib[8]	−62.2	±3.8	−28.3	±1.7
Gly[9](CS)	−89.2	±4.9	−52.2	±1.1
L-Ile[10]	–106.1	±2.8	−16.0	±3.1
L-Leu-OMe[11]	−49.1	±8.1	—	—

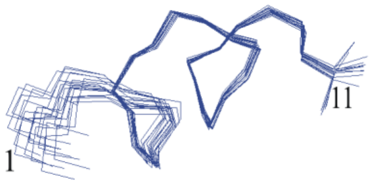

Figure S-I: Representation of the 18 3D structures with energy < 144 kcal/mol resulting from the MD calculations of **ψ[CS-NH][9]** with the backbone atoms of residues 4–11 superimposed.

Chemical characterization data for the peptides reported in Schemes 1–3 and not characterized elsewhere [1-5]

Boc-Aib-Gly-Ile-Leu-OMe: mp 170–172 °C; R_{f1} 0.40; R_{f2} 0.85; R_{f3} 0.30; $[\alpha]_D^{20}$: −91.1 (*c* 0.1, MeOH); ^{1}H NMR (200 MHz, $CDCl_3$) δ 7.86 (d, 1H, Ile NH), 7.00 (t, 1H, Gly NH), 6.77 (d, 1H, Leu NH), 5.02 (s, 1H, Aib NH), 4.64–4.52 (m, 1H, Leu α-CH), 4.34–4.27 (m, 1H, Ile α-CH), 3.95–3.27 (d, 2H, Gly α-CH_2), 3.70 (s, 3H, OMe CH_3), 2.07 (m, 1H, Ile β-CH), 1.78–1.63 (m, 20H, Leu γ-CH, Boc 3 CH_3, Aib 2 β-CH_3, Leu β-CH_2, Ile γ-CH_2), 0.95–0.85 (m, 12H, Leu 2 δ-CH_3, Ile γ- and δ-CH_3); IR (KBr) 3288, 1756, 1652, 1526 cm^{-1}.

Boc-Gly-Leu-Aib-Gly-Ile-Leu-OMe: mp 82–84 °C; R_{f1} 0.40; R_{f2} 0.95; R_{f3} 0.30; ^{1}H NMR (200 MHz, $CDCl_3$) δ 7.86–7.22 (d, 1H, Leu NH), 7.34–7.30 (m, 2H, Ile NH, Gly NH), 6.99 (s, 1H, Aib NH), 6.73–6.69 (d, 1H, Leu NH), 5.68–5.62 (t, 1H, Gly NH), 4.18–3.35 (m, 10H, 2 Leu α-CH, Ile α-CH, 2 Gly α-CH_2, OMe CH_3), 2.08 (m, 1H, Ile β-CH), 1.64–1.50 (m, 14H, 2 Leu β-CH_2, 2 Leu γ-CH, Aib 2 β-CH_3), 1.41 (s, 9H, Boc 3 CH_3, Ile γ-CH_2), 0.95–0.82 (m, 18H, 2 Leu 2 δ-CH_3, Ile γ- and δ-CH_3); IR (KBr): 3315, 1654, 1536 cm^{-1}.

Boc-Gly-ψ[CS-NH]-Leu-Aib-OMe: mp 79–82 °C; R_{f1} 0.90; R_{f2} 0.90; R_{f3} 0.45; $[\alpha]_D^{20}$ −58.8 (*c* 0.5, MeOH); ^{1}H NMR (200 MHz, $CDCl_3$) δ 8.97 (d, 1H, Leu NH), 7.08 (s,1H, Aib NH), 5.51 (m, 1H, Gly NH), 4.43 (m, 2H, Gly α-CH_2), 4.12 (m, 1H, Leu α-CH), 3.71 (s, 3H, OMe CH_3), 1.66 (m, 3H; Leu β-CH_2 and γ-CH), 1.51 (m, 6H, Aib 2 β-CH_3), 1.41 (s, 9H Boc 3 CH_3), 0.93 (d, 6H, Leu 2 δ-CH_3); IR (KBr): 3446, 3280, 1738, 1720, 1694, 1652, 1547, 1526 cm^{-1}.

Boc-Aib-Gly-ψ[CS-NH]-Leu-Aib-OMe: mp 175–178 °C; R_{f1} 0.85; R_{f2} 0.90; R_{f3} 0.20; $[\alpha]_D^{20}$ −40.9 (*c* 0.5, MeOH); ^{1}H NMR (200 MHz, $CDCl_3$) δ 8.97 (d, 1H, Leu NH) 7.08 (s,1H, Aib NH), 6.10 (s,1H, Aib NH), 5.51 (m, 1H, Gly NH), 4.43 (m, 2H, Gly α-CH_2), 4.12 (m, 1H, Leu α-CH), 3.71 (s, 3H, OMe CH_3), 1.66 (m, 3H; 2H Leu β-CH_2 and γ-CH), 1.51 (m, 12H, 2 Aib 2 β-CH_3), 1.41 (s, 9H Boc 3 CH_3), 0.93 (d, 6H, Leu 2 δ-CH_3); IR (KBr): 3349, 3265, 1717, 1691, 1665, 1510 cm^{-1}.

***n*-Oct-Aib-Gly-ψ[CS-NH]-Leu-Aib-OMe:** oil; R_{f1} 0.81; R_{f2} 0.90; R_{f3} 0.15; $[\alpha]_D^{20}$ −5.68 (*c* 0.2, MeOH); ^{1}H NMR (200 MHz, CDCl3) δ 8.97 (d, 1H, Leu NH) 7.08 (s, 1H, Aib NH), 6.10 (s, 1H, Aib NH), 5.51 (m, 1H, Gly NH), 4.43 (m, 2H, Gly α-CH_2), 4.12 (m, 1H, Leu α-CH), 3.71 (s, 3H, OMe CH_3), 2.28 (m, 2H, *n*-Oct β-CH_2), 1.66 (m, 3H, Leu β-CH_2 and γ-CH), 1.51 (m, 12H, 2 Aib 2 β-CH_3), 1.26 (m, 10H, *n*-Oct 5 CH_2), 0.93–0.82 (m, 9H, Leu 2 δ-CH_3 and *n*-Oct ω-CH_3); IR (KBr): 3313, 3240, 1742, 1730, 1650, 1531 cm^{-1}.

***n*-Oct-Aib-Gly-ψ[CS-NH]-Leu-Aib-OH:** R_{f1} 0.10; R_{f2} 0.70; R_{f3} 0.00; ^{1}H NMR (200 MHz, $CDCl_3$) δ 8.97 (d, 1H, Leu NH) 7.08 (s, 1H, Aib NH), 6.10 (s, 1H, Aib NH), 5.51 (m, 1H, Gly NH), 4.43 (m, 2H, Gly α-CH_2), 4.12 (m, 1H, Leu α-CH), 2.28 (m, 2H, *n*-Oct β-CH_2), 1.66 (m, 3H, Leu β-CH_2 and γ-CH), 1.51 (m, 12H, 2 Aib 2 β-CH_3), 1.26 (m, 10H, *n*-Oct 5 CH_2), 0.93–0.82 (m, 9H, Leu 2 δ-CH_3 and *n*-Oct ω-CH_3); IR (KBr): 3310, 3044,1720, 1650, 1531 cm^{-1}.

***n*-Oct-Aib-Gly-ψ[CS-NH]-Leu-Aib-Gly-Gly-Leu-Aib-Gly-Ile-Leu-OMe:** mp 114–116 °C; R_{f1} 0.20; R_{f2} 0.85; R_{f3} 0.00; $[\alpha]_D^{20}$ −38.61 (c 0.5, MeOH); ^{1}H NMR (400 MHz, CD_3CN) δ 9.30 (d, 1H, NH Leu^3), 7.77 (t, 1H, NH Gly^6), 7.49 (t, 1H, NH Gly^2), 7.47 (t, 1H, NH Gly^5), 7.47 (s, 1H, NH Aib^4), 7.42 (t, 1H, NH Gly^9), 7.40 (d, 1H, NH Leu^7), 7.37 (s, 1H, NH Aib^8), 7.30 (d, 1H, NH Ile^{10}), 7.22 (s, 1H, NH Aib^1), 7.12 (d, 1H, NH Leu^{11}), 4.21 (m, 1H, α-CH Leu^{11}), 4.20 (m, 1H, α-CH Leu^7), 4.17 (m, 1H, α-CH Leu^3), 4.05 (dd, 1H, α-CH Ile^{10}), 3.78 (dd, 1H, 1 α-CH Gly^6), 3.74 (dd, 1H, 1 α-CH Gly^6), 3.71 (s, 3H, CH_3 OMe), 3.67 (dd, 1H, 1 α-CH Gly^9), 3.65 (dd, 1H, 1 α-CH Gly^2), 3.64 (m, 2H, α-CH_2 Gly^5), 3.60 (dd, 1H, 1 α-CH Gly^9), 3.58 (dd, 1H, 1 α-CH Gly^2), 2.15 (t, 2H, β-CH_2 *n*-Oct), 1.50 (m, 2H, γ-CH_2 *n*-Oct), 2.15 (m, 2H, δ-CH_2 *n*-Oct), 1.84 (m, 1H, β-CH Ile^{10}), 1.53 (m, 2H, β-CH_2 Leu^7), 1.53 (m, 2H, β-CH_2 Leu^3), 1.62 (m, 1H, γ-CH Leu^3), 1.60 (m, 1H, γ-CH Leu^7), 1.59 (m, 1H, γ-CH Leu^{11}), 1.48 (m, 1H, 1 β-CH Leu^{11}), 1.53 (m, 1H, 1 β-CH Leu^{11}), 1.45 (m, 1H, γ-CH Ile^{10}), 1.36 (s, 6H, 2 β-CH_3 Aib^4), 1.35 (s, 6H, 2 β-CH_3 Aib^8), 1.32 (s, 6H, 2 β-CH_3 Aib^1), 1.17 (m, 1H, 1 γ-CH Ile^{10}), 0.88 (m, 3H, δ-CH_3 Leu^3), 0.87 (m, 3H, δ-CH_3 Leu^{11}), 0.86 (d, 3H, γ-CH_3 Ile^{10}), 0.83 (m, 3H, δ-CH_3 Leu^3), 0.81 (m, 3H, δ-CH_3 Ile^{10}), 0.80 (t, 3H, ω-CH_3 *n*-Oct), 0.78 (m, 3H, δ-CH_3 Leu^{11}); IR (KBr): 3314, 3057, 1744, 1656, 1537 cm^{-1}.

Z-Gly-Leu-Aib-O*t*-Bu: mp 112–114 °C; R_{f1} 0.80; R_{f2} 0.65; R_{f3} 0.50; $[\alpha]_D^{20}$ −39.0 (*c* 0.5, MeOH); ^{1}H NMR (200 MHz, $CDCl_3$) δ 7.33 (m, 5H, Z Ph), 6.85 (s, 1H, Aib NH), 6.79 (d,

1H, Leu NH), 5.70 (t, 1H, Gly NH), 5.10 (s, 2H, Z CH_2), 4.40 (m, 1H, Leu α-CH), 3.86 (t, 2H, Gly α-CH_2), 1.58 (m, 3H; Leu β-CH_2 and γ-CH), 1.51 (2s, 6H, Aib 2 β-CH_3), 1.43 (s, 9H, O*t*-Bu 3 CH_3), 0.91 (d, 6H, Leu 2 δ-CH_3); IR (KBr): 3324, 1731, 1705, 1659, 1546 cm^{-1}.

Z-Aib-Gly-Leu-Aib-O*t*-Bu: mp 165–166 °C; R_{f1} 0.50; R_{f2} 0.80; R_{f3} 0.30; $[\alpha]_D^{20}$ −3.2 (*c* 0.5, MeOH); ^{1}H NMR (200 MHz, $CDCl_3$) δ 7.36 (m, 6H, Z Ph and 1 NH), 6.93 (m, 1H, NH), 6.87 (s, 1H, Aib NH), 5.37 (s, 1H, Aib NH), 5.10 (2d, 2H, Z CH_2), 4.42 (m, 1H, Leu α-CH), 3.93 (d, 2H, Gly α-CH_2), 1.70–1.68 (m, 3H, Leu β-CH_2 and γ-CH), 1.50–148 (m, 12H, 2 Aib 2 β-CH_3), 1.42 (s, 9H, O*t*-Bu 3 CH_3), 0.91 (m, 6H, 2 Leu δ-CH_3); IR (KBr): 3316, 1732, 1701, 1659, 1536 cm^{-1}.

***n*-Oct-Aib-Gly-Leu-Aib-O*t*-Bu:** mp 169–171 °C; R_{f1} 0.45; R_{f2} 0.55; R_{f3} 0.30; $[\alpha]_D^{20}$ −2.58 (*c* 0.5, MeOH); ^{1}H NMR (200 MHz, $CDCl_3$) δ 6.93 (m, 1H, NH), 6.87 (s, 1H, Aib NH), 5.37 (s, 1H, Aib NH), 4.42 (m, 1H, Leu α-CH), 3.93 (d, 2H, Gly α-CH_2), 2.23 (m, 2H *n*-Oct β-CH_2) 1.70–1.68 (m, 3H, Leu β-CH_2 and γ-CH), 1.50–1.48 (m, 12H, 2 Aib 2 β-CH_3), 1.42 (s, 9H, O*t*-Bu 3 CH_3), 1.26 (m, 10H, *n*-Oct 5 CH_2), 0.95–0.82 (m, 6H, 2 Leu δ-CH_3, *n*-Oct ω-CH_3); IR (KBr): 3315, 1740, 1709, 1656, 1536 cm^{-1}.

Fmoc-Gly-ψ[CS-NH]-Gly-Leu-Aib-O*t*-Bu: mp 108–111 °C; R_{f1} 0.72; R_{f2} 0.94; R_{f3} 0.22; $[\alpha]_D^{20}$ −18.6 (*c* 0.5, MeOH); ^{1}H NMR (200 MHz, $CDCl_3$) δ 8,98 (t, 1H, Gly NH), 7.75 (d, 2H, Fmoc), 7.58 (t, 2H, Fmoc), 7.49 (m, 2H, Fmoc), 6.81 (s, 1H, Aib NH), 6.67 (t, 1H, Leu NH), 5.99 (t, 1H, Gly NH), 5.02 (m, H, Leu α-CH), 4.61–4.73(m,3H, Fmoc), 3.95–3.27 (d, 4H, 2Gly α-CH_2), 1.63–1.78(m, 9H, Leu β-CH_2 and γ-CH, Aib 2 β-CH_3), 1.43 (s, 9H, O*t*-Bu 3 CH_3), 0.92 (m, 6H, Leu 2 δ-CH_3); IR (KBr): 3299, 1730, 1706, 1658, 1528 cm^{-1}.

Fmoc-Gly-ψ[CS-NH]-Gly-Leu-Aib-Gly-Ile-Leu-OMe: mp 126–128 °C; R_{f1} 0.74; R_{f2} 0.92; R_{f3} 0.15; $[\alpha]_D^{20}$ –17.3 (*c* 0.5, MeOH); ^{1}H NMR (200 MHz, $CDCl_3$) δ 8.98 (t, 1H, Gly NH), 7.86 (d, 1H, Ile NH), 7.75 (d, 2H, Fmoc), 7.58 (t, 2H, Fmoc), 7.49 (m, 2H, Fmoc), 7.00 (t, 1H, Gly NH), 6.81 (s, 1H, Aib NH), 6.67 (m, 2H, 2 Leu NH), 5.99 (t, 1H, Gly NH), 5.02 (m, 2H, 2 Leu α-CH), 4.62–4.87 (m, 3H, Fmoc), 4.34–4.27 (m, 1H, Ile α-CH), 3.95–3.27 (d, 6H, 3Gly α-CH_2), 2.07 (m, 1H, Ile β-CH), 1.78–1.63 (m, 14H, 2 Leu β-CH_2 and γ-CH, Aib 2 β-CH_3, Ile γ-CH_2), 1.43 (s, 9H, O*t*-Bu 3 CH_3), 0.95–0.85 (m, 24H, 2 Leu 2 δ-CH_3, Ile γ-CH_3 and δ-CH_3); IR (KBr): 3309, 3065, 1730, 1656, 1531cm^{-1}.

***n*-Oct-Aib-Gly-Leu-Aib-Gly-ψ[CS-NH]-Gly-Leu-Aib-Gly-Ile-Leu-OMe:** mp 125–128 °C; R_{f1} 0.20; R_{f2} 0.85; R_{f3} 0.00; $[\alpha]_D^{20}$ −21.3 (*c* 0.5, MeOH); ^{1}H NMR (200 MHz, CD_3CN) δ 9.42 (t, 1H, NH Gly^{6}), 7.75 (t, 1H, NH Gly^{5}), 7.67 (d, 1H, NH Leu^{3}), 7.540 (s, 1H, NH Aib^{4}), 7.40 (t, 1H, NH Gly^{2}), 7.33 (t, 1H, NH Gly^{9}), 7.29 (d, 1H, NH Ile^{10}), 7.28 (d, 1H, NH Leu^{7}), 7.27 (s, 1H, NH Aib^{8}), 7.17 (s, 1H, NH Aib^{1}), 7.07 (d, 1H, NH Leu^{11}), 4.21 (m, 1H, α-CH Leu^{11}), 4.20 (m, 1H, α-CH Leu^{7}), 4.17 (m, 1H, α-CH Leu^{3}), 4.05 (dd, 1H, α-CH Ile^{10}), 3.78 (dd, 1H, 1 α-CH Gly^{6}), 3.74 (dd, 1H, 1 α-CH Gly^{6}), 3.71 (s, 3H, CH_3 OMe), 3.67 (dd, 1H, 1 α-CH Gly^{9}), 3.65 (dd, 1H, 1 α-CH Gly^{2}), 3.64 (m, 2H, α-CH Gly^{5}), 3.60 (dd, 1H, 1 α-CH Gly^{9}), 3.58 (dd, 1H, 1 α-CH Gly^{2}), 2.15 (t, 2H, β-CH_2 *n*-Oct), 2.15 (m, 2H, δ-CH_2 *n*-Oct), 1.84 (m, 1H, β-CH Ile^{10}), 1.68 (m, 1H, γ-CH Leu^{3}), 1.60 (m, 1H, γ-CH Leu^{7}), 1.59 (m, 1H, γ-CH Leu^{11}), 1.53 (m, 1H, 1 β-CH Leu^{11}), 1.53 (m, 2H, β-CH_2 Leu^{7}), 1.53 (m, 2H, β-CH_2 Leu^{3}), 1.50 (m, 2H, γ-CH_2 *n*-Oct), 1.48 (m, 1H, 1 β-CH Leu^{11}), 1.47 (m, 1H, γ-CH_2 Ile^{10}), 1.46 (s, 6H, 2 β-CH_3 Aib^{4}), 1.35 (s, 6H, 2 β-CH_3 Aib^{8}), 1.32 (s, 6H, 2 β-CH_3 Aib^{1}), 1.28 (m, 1H, 1 γ-CH_2 Ile^{10}), 0.88 (m, 3H, δ-CH_3 Leu^{3}), 0.87 (m, 3H, δ-CH_3 Leu^{11}), 0.86 (d, 3H, γ-CH_3 Ile^{10}), 0.83 (m, 3H, δ-CH_3 Leu^{3}), 0.81 (m, 3H, δ-CH_3 Ile^{10}), 0.80 (t, 3H, ω-CH_3 *n*-Oct), 0.78 (m, 3H, δ-CH_3 Leu^{11}); IR (KBr): 3302, 1742, 1657, 1651, 1536 cm^{-1}.

Boc-Gly-ψ[CS-NH]-Ile-Leu-OMe: oil; R_{f1} 0.90; R_{f2} 0.92; R_{f3} 0.60; $[\alpha]_D^{20}$ −56.1 (*c* 0.5, MeOH); ^{1}H NMR (200 MHz, $CDCl_3$) δ 8.74 (d, 1H, Ile NH), 6.53–6.48 (d, 1H, Leu NH), 5.45–5.37 (t, 1H, Gly NH), 4.62–3.76 (m, 4H, Ile αCH, Leu αCH, Gly αCH_2), 3.71 (s, 3H, OMe CH_3), 2.20–1.46 (m, 6H, Ile β-CH and γ-CH_2, Leu β-CH_2 and γ-CH), 1.44 (s, 9H, Boc 3 CH_3), 1.00–0.74 (m, 12H, Leu 2 δ-CH_3, Ile γ-CH_3 and δ-CH_3); IR (KBr): 3313, 1747, 1721, 1659, 1594, 1524 cm^{-1}.

***n*-Oct-Aib-Gly-Leu-Aib-Gly-Gly-Leu-Aib-Gly-ψ[CS-NH]-Ile-Leu-OMe:** oil; R_{f1} 0.20; R_{f2} 0.87; R_{f3} 0.00; $[\alpha]_D^{20}$ −23.3 (*c* 0.2, MeOH); ^{1}H NMR (400 MHz, CD_3CN) δ 8.96 (d, 1H, NH Ile^{10}), 7.91(t, 1H, NH Gly^{6}), 7.84 (t, 1H, NH Gly^{5}), 7.74 (d, 1H, NH Leu^{3}), 7.69 (t, 1H, NH Gly^{2}), 7.66 (t, 1H, NH Gly^{9}), 7.60 (d, 1H, NH Leu^{7}), 7.540 (s, 1H, NH Aib^{4}), 7.52 (s, 1H, NH Aib^{8}), 7.35 (s, 1H, NH Aib^{1}), 7.13 (d, 1H, NH Leu^{11}), 4.21 (m, 1H, α-CH Leu^{11}), 4.20 (m, 1H, α-CH Leu^{7}), 4.17 (m, 1H, α-CH Leu^{3}), 4.05 (dd, 1H, α-CH Ile^{10}), 3.78 (dd, 1H, 1 α-CH Gly^{6}), 3.74 (dd, 1H, 1 α-CH Gly^{6}), 3.71(s, 3H, CH_3 OMe), 3.68 (dd, 1H, 1 α-CH Gly^{9}), 3.66 (dd, 1H,

1 α-CH Gly[2]), 3.64 (m, 2H, α-CH_2 Gly[5]), 3.60 (dd, 1H, 1 α-CH Gly[9]), 3.58 (dd, 1H, 1 α-CH Gly[2]), 2.25 (t, 2H, β-CH_2 *n*-Oct), 1.57 (m, 2H, γ-CH_2 *n*-Oct), 2.15 (m, 2H, δ-CH_2 *n*-Oct), 1.84 (m, 1H, β-CH Ile[10]), 1.53 (m, 2H, β-CH_2 Leu[7]), 1.53 (m, 2H, β-CH_2 Leu[3]), 1.68 (m, 1H, γ-CH Leu[3]), 1.60 (m, 1H, γ-CH Leu[7]), 1.59 (m, 1H, γ-CH Leu[11]), 1.48 (m, 1H, 1 β-CH Leu[11]), 1.53 (m, 1H, 1 β-CH Leu[11]), 1.47 (m, 1H, γ-CH Ile[10]), 1.46 (s, 6H, 2 β-CH_3 Aib[4]), 1.35 (s, 6H, 2 β-CH_3 Aib[8]), 1.32 (s, 6H, 2 β-CH_3 Aib[1]), 1.28 (m, 1H, 1 γ-CH Ile[10]), 0.88 (m, 3H, δ-CH_3 Leu[3]), 0.86 (m, 3H, δ-CH_3 Leu[11]), 0.84 (d, 3H, γ-CH_3 Ile[10]), 0.83 (m, 3H, δ-CH_3 Leu[3]), 0.82 (m, 3H, δ-CH_3 Ile[10]), 0.79 (t, 3H, ω-CH_3 *n*-Oct), 0.78 (m, 3H, δ-CH_3 Leu[11]); IR (KBr): 3299, 1741, 1660, 1537 cm^{-1}.

References

1. De Zotti, M.; Biondi, B.; Peggion, C.; Formaggio, F.; Park, Y.; Hahm, K.-S.; Toniolo, C. *Org. Biomol. Chem.* **2012,** *10,* 1285–1299. doi:10.1039/c1ob06178j
2. Gurunath, R.; Balaram, P. *Biopolymers* **1995,** *35,* 21–29. doi:10.1002/bip.360350104
3. Iida, A.; Yoshimatsu, S.; Sanekata, M.; Fujita, T. *Chem. Pharm. Bull.* **1990,** *38,* 2997–3003. doi:10.1248/cpb.38.2997
4. Brückner, H.; Koza, A. *Amino Acids* **2003,** *24,* 311–323. doi:10.1007/s00726-002-0401-x
5. Nagaraj, R.; Balaram, P. *Tetrahedron* **1981,** *37,* 1263–1270. doi:10.1016/S0040-4020(01)92061-7
